# An Immunocompetent Adult Male Presented with a Liver Abscess Caused by the *Mycobacterium tuberculosis*: A Case Report from India

**DOI:** 10.1155/2023/9049315

**Published:** 2023-08-14

**Authors:** Akash Pawar, Sagar Khadanga, Abhishek Singhai

**Affiliations:** ^1^Department of Trauma & Emergency Medicine, All India Institute of Medical Sciences, Bhopal, India; ^2^Department of General Medicine, All India Institute of Medical Sciences, Bhopal, India

## Abstract

A liver abscess is a collection of purulent fluid in the liver parenchyma caused by a variety of etiological organisms such as bacteria, protozoa, and in rare occasions fungi. *Mycobacterium tuberculosis* (MTB) is a frequent and widespread infection in underdeveloped nations such as India, which can manifest in many ways. Tuberculosis mostly affects the lungs, although it can also affect any organ in the body. We are reporting a case of liver abscess caused by MTB infection in order to raise general awareness among physicians about the importance of suspecting and ruling out tuberculosis as a cause of liver abscess. To the best of our knowledge, there have been very few such cases reported from India/the rest of the world.

## 1. Introduction

A liver abscess is an accumulation of pus in the liver caused by direct liver infection through systemic circulation or an intraabdominal infection disseminated by the portal vein [1]. Liver abscesses are generally classified as pyogenic or amoebic, although fungi and parasites have a role in a small number of cases. Even though the prevalence of liver abscess is minimal, it is important to suspect, evaluate, and treat these lesions as early as possible since untreated patients face a considerable mortality risk. 50% of solitary hepatic abscesses occur in the right lobe of the liver (a larger portion with higher blood supply) [2, 3].

Tuberculosis caused by *Mycobacterium tuberculosis* (MTB) which is anacid fast bacilli is a prevalent health problem in India. It can manifest in many ways and affect nearly all organs in the human body [4]. The involvement of the liver in pulmonary and extrapulmonary tuberculosis is frequently clinically silent. Primary or local hepatic tuberculosis in the absence of current pulmonary or miliary tuberculosis is a rare diagnosis [5]. It mimics hepatic abscess due to other etiology, and the diagnosis is frequently missed due to a lack of suspicion. We present a case of tubercular liver abscess (confirmed by a cartridge-based nucleic acid amplification test for MTB) in a 48-year-old male patient with no known comorbidities and no involvement of tuberculosis infection in any other part of the body. This case stresses the need of suspecting, evaluating, and ruling out tuberculosis as an etiological agent in patients with liver abscesses because it is a curable disease, with rapid diagnosis and treatment can avert serious consequences.

## 2. Case Presentation

48-year-old male with no known comorbidities presented with complaint of abdominal pain, low-grade fever, loss of appetite, and weight since last 1 month. Ultrasound abdomen of this patient was suggestive of multiple abscesses in the left lobe of the liver, the largest measuring 4.5 × 4.8 × 3.6 cm, for which the patient had received various treatments at multiple health centers over the past month including injectable and oral antibiotics.

**Figure 1:**
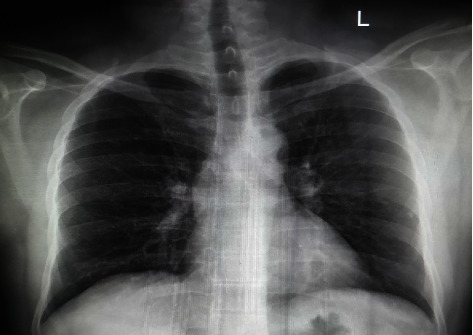
Chest x-ray of the patient.

The patient was subsequently admitted in our hospital and thoroughly evaluated which includes a detailed history, clinical examination, biochemical, pathological, microbiological tests, and radiological imaging (Table 1). The significant laboratory and radiological finding were anemia, low albumin, high CRP, normal chest x-ray (Figure 1), and abdominal ultrasonography suggestive of identical sized abscesses in the left lobe of the liver and computed tomography of the abdomen suggestive of multiple liver abscesses in the left lobe with no other abnormalities, and then the patient underwent ultrasound guided pus aspiration and aspirate sent for fluid analysis.

The fluid cytology showed that fluid was suppurative with primarily degraded, viable polymorphs, acid fast bacilli in a necrotic backdrop. The cartridge-based nucleic acid amplification test (CBNAAT) for MTB of the aspirated sample detected MTB that was rifampicin-sensitive. Patient was then started on antitubercular treatment as per the standard National Tuberculosis Elimination Program guidelines for tuberculosis according to weight (patient's weight-52 kg) which comprises four 1^st^ line drugs for a duration of 6 months (details in Table 2) [6] and discharged with regular follow-up in the medicine outpatient department. During follow-up, he responded well to the treatment.

## 3. Discussion

The liver is one of the organs that is more prone to develop a parenchymal abscess, particularly in the right lobe of the liver [2], and different etiological agents have been identified as the cause of liver abscess. MTB is also one of the causes of liver abscess, but it is uncommon, especially in immunocompetent adults with isolated tubercular liver abscess in the left lobe (no involvement of any other part) [5].

In this case report, the patient was not relieved by the routine antibiotics treatment, so we decided to further evaluate him to determine the cause and treat him accordingly. During the evaluation, tuberculosis infection was identified as a cause of liver abscess in this patient. To avoid local and systemic significant consequences, it is necessary to detect and evaluate for MTB as a causative agent for liver abscess. Similar cases have been reported before by several authors, including Yazan et al. in 2021 [7] and Devi et al. in 2019 [8], who likewise concluded that isolated tubercular liver abscess is a rare condition. However, it should be considered as differential diagnosis in endemic areas.

When we consider prevalence, prognosis, and complications among various etiologies of liver abscess (such as amoebic, pyogenic, and tuberculosis), amoebic liver abscess is the most common, whereas pyogenic/tubercular liver abscess is less common with poorer prognosis and more complications as compared to amoebic liver abscess [9]. Our reported case improved significantly by treatment with no sequential complication. Tubercular liver abscess has a fair prognosis, especially if detected and treated early. Suspecting and evaluating for *Mycobacterium tuberculosis* as a cause of any symptoms/disease (liver abscess in this case) should not be a “out of the box” strategy but rather an “in the box” approach in areas where tuberculosis is prevalent/endemic.

Nontuberculous mycobacteria (NTM) infection is a mycobacterium infection that can involve the same organs as MTB. NTM infection has similar smear positive to MTB, making it a difficult condition to evaluate and differentiate. In the previous studies, Dumic et al. in 2021 [10] and Gopalaswamy et al. in 2020 [11] found that adequate source control is a successful treatment option for rapidly growing NTM infection in immunocompetent patients in the absence of antimicrobial therapy, which is not the case in MTB infection.

## 4. Conclusion

Tubercular liver abscess is a unique and uncommon diagnosis. The infection mimics abscess by other etiologies, and the diagnosis is sometimes missed due to a lack of suspicion. As a result, our goal in reporting this case is to raise awareness among health professionals about the importance of including tuberculosis in the differential diagnosis of mass or cystic/abscess lesions of the liver, especially in a nation with a high tuberculosis prevalence, and to evaluate/rule out the condition on a regular basis.

## Figures and Tables

**Table 1:** Laboratory parameters and radiological imaging reports of the patient.

Serial no.	Investigations	Findings	Remarks
1	Haemoglobin	10.2 gm/dl	Low
2	White blood cells	4250/mm^3^	Normal
3	Platelets	2.10 lac/mm^3^	Normal
4	Serum CRP	48 mg/L	High
5	Serum urea	29.07 mg/dL	Normal
6	Serum creatinine	0.86 mg/dL	Normal
7	Alanine transaminase	13 U/L	Normal
8	Aspartate minotransferase	23 U/L	Normal
9	Total bilirubin	0.34 mg/dL	Normal
10	Serum albumin	2.72 g/dL	Low
11	INR	1.1	Normal
12	Random blood sugar	136 mg/dL	Normal
13	HIV/HBsAg/Anti-HCV	Negative	Normal
14	Blood culture	Sterile	Normal
15	Aspiration fluid/puscytology	Suppurative	Lymphocytic rich, AFB-positive
16	Fluid/pus CBNAAT	MTB-detected	RIF-sensitive
17	Chest x-ray (Figure 1)	No abnormalities	Normal
18	Ultrasonography (abdomen)	Multiple liver abscess in the left lobe	Largest measuring: 200 ml
19	Computed tomography of the chest and abdomen	Multiple liver abscess in the left lobe	Intestines normal and no lymphadenopathy and no any other abnormalities

**Table 2:** Treatment details [6].

Serial no.	Weight category (Total body weight in kgs)	Tablets	
		Intensive phase (IP) (2 months) H/R/Z/E (in mg) 75/150/400/275	Continuation phase (CP) (4 months) H/R/E (in mg) 75/150/275
1	25 to 39	2 tablets	2 tablets
2	40 to 54	3 tablets	3 tablets
3	55 to 69	4 tablets	4 tablets
4	≥70	5 tablets	5 tablets

H: isoniazid, R: rifampicin, Z: pyrazinamide, and E: ethambutol. The patient weighed 52 kg and was started on 4 drugs' regimen for 2 months (IP), 4 tablets per day, and then 3 drugs' regimen for 4 months (CP).

## Data Availability

The patient data used to support the findings of this study are included within the article.
